# Complete chloroplast genome of *Isoetes hypsophila* (Isoetaceae), the Endangered quillwort in China

**DOI:** 10.1080/23802359.2021.1960216

**Published:** 2021-09-13

**Authors:** Yufeng Gu, Junhao Yu, Hui Shang, Baodong Liu, Yuehong Yan

**Affiliations:** a Key Laboratory of National Forestry and Grassland Administration for Orchid Conservation and Utilization, The National Orchid Conservation & Research Center of Shenzhen, Shenzhen, Guangdong, China; b Key Laboratory of Plant Biology in Colleges of Heilongjiang Province, Life Science and Technology College, Harbin Normal University, Harbin, Heilongjiang, China; c College of Life Science, Shanghai Normal University, Shanghai, China; d Shanghai Chenshan Plant Science Research Center, Chinese Academy of Sciences, Shanghai, China

**Keywords:** *Isoetes hypsophila*, plastome, plateau wetland

## Abstract

*Isoetes hypsophila* Hand.-Mazz. is an Endangered quillwort living in plateau wetlands in China. In the present study, the complete chloroplast genome of *I*. *hypsophila* was assembled. It is a circular form of 146,362 bp in length, comprising a pair of inverted repeat (IR) regions of 13,691 bp, a large single copy (LSC) of 91,741 bp, and a small single copy (SSC) of 27,239 bp. After annotation, a total of 135 genes were predicted, which are 84 encode proteins 37 tRNA and 8 rRNA. The phylogenetic analysis indicated that *I*. *hypsophila* clustered with a clade of *I*. *sinensis*, *I*. *taiwanensis* and *I*. *yunguiensis* with strong support value. The chloroplast genome will contribute to further research and conservation of *I*. *hypsophila*.

*Isoetes hypsophila* Hand.-Mazz. is a member of Isoetaceae, only distributing in Yunnan and Sichuan Province in China (Chen et al. 2005; Zhang and Taylor 2013). This species grows in the plateau wetlands with altitude more than 3,700 m. The height of *I*. *hypsophila* is always less than 10 centimeters, and its megaspore is smooth (Liu et al. 2008), which make it easy to distinguish from other species in China. The chromosome number of *I*. *hypsophila*is 2n = 22 (Liu et al. 2002). Here, we assembled the complete chloroplast genome of *I*. *hypsophila* to provide genomic resources for conservation and breeding of this taxon.

In this study, fresh leaves of *I*. *hypsophila* were aquired from Sangdui Village, Daocheng County, Sichuan Province, China (100°3′41.81″E and 29°8′44.09″N) and then dried with silica. Specimens (voucher: Yufeng Gu & Junhao Yu Fern08963) were deposited at Herbarium of National Orchid Conservation & Research Center of Shenzhen (NOCC!). We sent silica-dried material to the Shanghai Majorbio Bio-pharm Technology Co., Ltd (Shanghai, China), and the sequencing was taken on an Ilumina Hiseq × Ten platform (Illumina, San Diego, CA, USA). Plastid genome assembled using GetOrganelle v1.7.5 (Jin et al. 2018), and the results were viewed and edited by Bandage v0.8.1 (Wick et al. 2015). Assembled chloroplast genome was annotated by Geneious Prime 2021.0.3 (Free Trial) (Kearse et al. 2012) with *I*. *engelmannii* as reference. The annotated chloroplast geome was drawn with the online tool OGDRAW (http://ogdraw.mpimp-golm.mpg.de/) (Lohse et al. 2013).

The complete plastid genome sequence of *I*. *hypsophila* (GenBank accession no. MW405450) was 146,362 bp in length, containing a large single-copy (LSC) region of 91,741 bp, a small single-copy (SSC) region of 27,239 bp, and a pair of inverted repeats (IR) regions of 13,691 bp. A total of 135 gene species were annotated, including 84 protein-coding genes, 37 transfer RNA (tRNA) genes, and 8 ribosomal RNA (rRNA) genes. The complete genome GC content was 38.10%.

To figure out the phylogenetic position of *I*. *hypsophila,* the molecular phylogenetic analysis was carried out with 15 published complete chloroplast genomes of *Isoetes* downloaded from GenBankand three members of Lycopodiaceae (*Lycopodium clavatum*, *Huperzia serrata* and *H*. *lucidula*) set as outgroup. The sequences were aligned using MAFFT v7 in PhyloSuite v1.2.2 (Zhang et al. 2020). The maximum-likelihood (ML) tree was constructed using RAxML v8 under the GTRCAT nucleotide substitution model and 1,000 rapid bootstraps (Stamatakis 2014).

The ML tree indicated that *Isoetes* is monophyletic. *I*. *serracarajensis* and *I*. *cangae* form a clade as sister group to all other species in this genus. *I*. *nuttallii* and *I*. *malinverniana* are two clades solely. *Isoetes hypsophila* was sister to the clade of *I*. *sinensis*, *I*. *yunguiensis*, and *I*. *taiwanensis* with 85% bootstrap support values (Figure 1). These fourChinese species form a clade as sister group to the clade comprising with *I*. *butleri*, *I*. *engelmannii*, *I*. *flaccida*, *I*. *graniticola*, *I*. *piedmontana*, *I*. *mattaponica*, *I*. *melanospora*, and *I*. *valida* with a the 100% support value.

**Figure 1.**
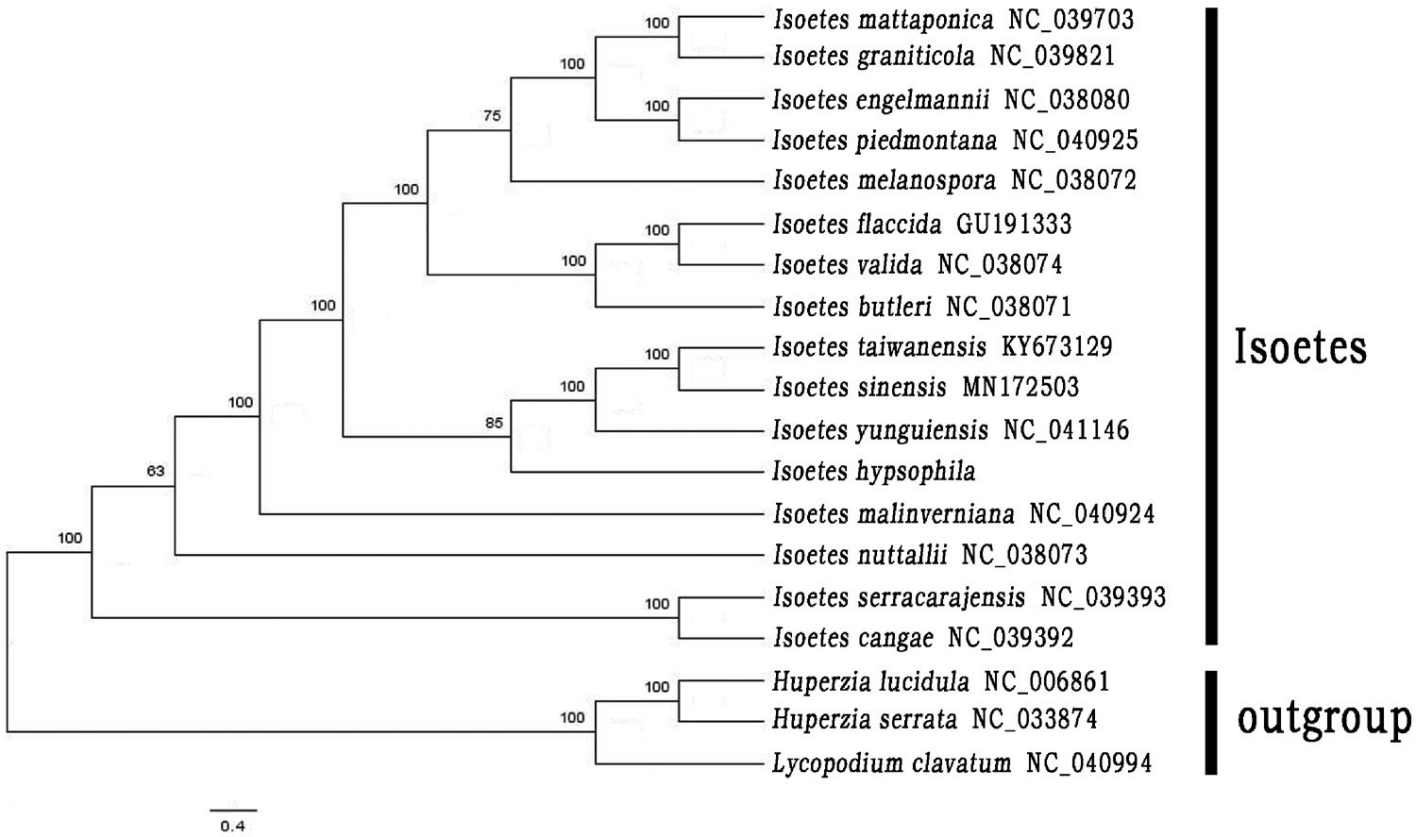
Maximum likelihood phylogenetic tree of 16 species of *Isoetes* and 3 taxa (*Lycopodium clavatum*, *Huperzia serrata* and *H*. *lucidula*) as outgroup based on plastid genome sequences by RAxML. The number on each node indicates bootstrap support value.

*Isoetes hypsophila* may be the ancestral species in China even Asia (Kim et al. 2010), the chloroplast genome will contribute to further research and the conservation of *I*. *hypsophila*.

## Data Availability

The genome sequence data that support the findings of this study can be obtained from GenBank of NCBI (https://www.ncbi.nlm.nih.gov/) under the accession no. MW405450.1. The associated Bioproject, SRA and Bio-sample numbers are PRJNA731595, SSR14618281 and SAMN19291068, respectively.
